# Prenatal treatment with rosiglitazone attenuates vascular remodeling and pulmonary monocyte influx in experimental congenital diaphragmatic hernia

**DOI:** 10.1371/journal.pone.0206975

**Published:** 2018-11-12

**Authors:** Jan-Hendrik Gosemann, Florian Friedmacher, Alejandro Hofmann, Julia Zimmer, Joachim F. Kuebler, Susanne Rittinghausen, Anne Suttkus, Martin Lacher, Luis Alvarez, Nicolae Corcionivoschi, Prem Puri

**Affiliations:** 1 National Children’s Research Centre, Our Lady’s Children’s Hospital, Dublin, Ireland; 2 Department of Pediatric Surgery, University of Leipzig, Leipzig, Germany; 3 The Royal London Hospital, London, United Kingdom; 4 Department of Pediatric Surgery, Hannover Medical School, Hannover, Germany; 5 Fraunhofer Institute for Toxicology and Experimental Medicine ITEM, Hannover, Germany; 6 Wellcome Centre Human Genetics, University of Oxford, Oxford, United Kingdom; 7 Agri-Food and Biosciences Institute, Belfast, Northern Ireland, United Kingdom; 8 School of Medicine and Medical Science and Conway Institute of Biomedical Research, University College Dublin, Dublin, Ireland; Vanderbilt University Medical Center, UNITED STATES

## Abstract

**Introduction:**

Extensive vascular remodeling causing pulmonary hypertension (PH) represents a major cause of mortality in patients with congenital diaphragmatic hernia (CDH). The chemokine monocyte chemoattractant protein-1 (MCP-1) is a biomarker for the severity of PH and its activation is accompanied by pulmonary influx of monocytes and extensive vascular remodeling. MCP-1 activation can be reversed by application of rosiglitazone (thiazolidinedione). We performed this study to evaluate the role of MCP-1 for the pathogenesis of PH in experimental CDH. We hypothesized that vascular remodeling and MCP-1 activation is accompanied by pulmonary influx of fetal monocytes and can be attenuated by prenatal treatment with rosiglitazone.

**Methods:**

In a first set of experiments pregnant rats were treated with either nitrofen or vehicle on gestational day 9 (D9). Fetal lungs were harvested on D21 and divided into CDH and control. Quantitative real-time polymerase chain reaction, Western blot (WB), and immunohistochemistry (IHC) were used to evaluate MCP-1 expression, activation, and localization. Quantification and localization of pulmonary monocytes/macrophages were carried out by IHC.

In a second set of experiments nitrofen-exposed dams were randomly assigned to prenatal treatment with rosiglitazone or placebo on D18+D19. Fetal lungs were harvested on D21, divided into control, CDH+rosiglitazone, and CDH+placebo and evaluated by WB as well as IHC.

**Results:**

Increased thickness of pulmonary arteries of CDH fetuses was accompanied by increased systemic and perivascular MCP-1 protein expression and significantly higher amounts of pulmonary monocytes/macrophages compared to controls (p<0.01). These effects were reversed by prenatal treatment with rosiglitazone (p<0.01 vs. CDH+P; control).

**Conclusion:**

Prenatal treatment with rosiglitazone has the potential to attenuate activation of pulmonary MCP-1, pulmonary monocyte influx, and vascular remodeling in experimental CDH. These results provide a basis for future research on prenatal immunomodulation as a novel treatment strategy to decrease secondary effects of PH in CDH.

## Introduction

The mortality of neonates with congenital diaphragmatic hernia (CDH) remains high despite recent advances in intensive care including extracorporeal membrane oxygenation, nitric oxide and alternative treatment strategies with sildenafil, bosentan and prostacyclins [1–5]. Persistent pulmonary hypertension (PH)–a result of perinatal vascular remodeling–is one of the main contributors to mortality in CDH [6]. To evaluate alternative treatment strategies, several groups investigated vascular changes in CDH lungs [6]. Although various pathomechanisms have been suggested, the molecular background of extensive vascular remodeling in CDH leading to PH remains elusive.

Monocyte chemoattractant protein-1 (MCP-1) is a powerful monocyte chemoattractant that has been found to be markedly increased in patients with PH and has therefore been suggested as a biomarker for the severity of PH [7,8]. MCP-1 activation and the subsequent pro-inflammatory phenotype can be dampened by application of the thiazolidinedione rosiglitazone, which has originally been developed as an insulin-sensitizer for the treatment of type 2 diabetes [9]. Due to its anti-inflammatory properties, rosiglitazone has been shown to inhibit MCP-1 expression, subsequent monocyte activation and ultimately to attenuate experimental pulmonary hypertension in rodent models [9–14].

To date, the effects of increased systemic MCP-1 expression in CDH remain unclear. Furthermore, protein localization and the biological activity of the chemoattractant have not been determined.

In this study we hypothesized that MCP-1 activation is accompanied by increased pulmonary influx of monocytes/macrophages, leading to vascular remodeling and contributing to the development of PH in a well-established rat model of CDH. We further aimed to investigate if prenatal attenuation of MCP-1 expression decreases pulmonary monocyte influx in CDH and therefore might represent a potential therapeutic target for the treatment of PH in CDH.

## Methods

### Animals and drug administration

Pathogen-free Sprague-Dawley rats (Harlan UK Ltd., Bicester, UK) were kept in a temperature-controlled environment with a 12h-light period and water and food *ad libitum*. In a first set of experiments, adult Sprague-Dawley rats were naturally mated overnight, separated and checked for plugging. Presence of spermatozoids in the vaginal smear was considered as proof of pregnancy and pregnant females were randomly divided into 2 groups (“control” and “CDH”). On gestational day 9 (D9), 100 mg of nitrofen (2,4-dichloro-*p*-nitrophenyl ether, WAKO Chemicals, Osaka, Japan) dissolved in 1 ml olive oil was administered intragastrically to animals of the CDH group, as previously described [15]. Animals assigned to the control group received vehicle only. Gestational day 21 (D21) was selected as endpoint and fetuses were delivered via cesarean section. Therefore, dams were anesthetized in an induction chamber using 2% isoflurane (Piramal Healthcare, Morpeth, UK) and maintained at 1% for delivery. Fetuses were euthanatized by decapitation and inspected by laparatomy for CDH. Only fetuses with left-sided CDH were included in the following analyses. Fetal serum was obtained by decapitation and exsanguination. After centrifugation (2000G for 5 min), supernatant was collected, snap frozen and stored at -80°C for further analysis. Left lungs from animals with CDH (n = 32) as well as lungs from controls (n = 32) were dissected and either snap-frozen (and stored native at -80°C) for RNA isolation and protein extraction or fixed in formalin for immunohistochemistry.

In a second set of experiments, timed-pregnant Sprague-Dawley rats were randomly assigned to one of three groups [control, CDH+Placebo (CDH+P) or CDH+Rosiglitazone (CDH+R)]. On D9, animals of the control group were administered vehicle only, whereas animals of the groups CDH+P and CDH+R received nitrofen as described above. Additionally, on D18 and D19, 3 mg/kg bodyweight of the thiazolidinedione rosiglitazone (Cayman Chemical, Ann Harbor, MI, USA) was administered intraperitoneally as single shot (in 100μL cottonseed oil + 400μL PBS) under isoflurane anesthesia to animals of the CDH+R group, while animals of the CDH+P group received diluent alone. The time point and dosage of prenatal rosiglitazone treatment were adopted from the previously described prenatal application of rosiglitazone in a model of hyperoxia-induced neonatal rat lung injury [11]. According to experimental set 1, dams were anesthetized on D21 and fetuses were delivered and processed for further examination as described (control: n = 15; CDH+P: n = 14; CDH+R: n = 16). All animal procedures were performed in accordance to the guidelines for management and welfare of laboratory animals and were approved by the Department of Health and Children (Ref: B100/4378) under the Cruelty to Animals Act, 1876, as amended by the European Council Directive of 2010 (2010/63/EU).

### RNA isolation and quantitative real-time polymerase chain reaction (qRT-PCR)

After dissection, total RNA was isolated from left fetal lungs using the acid guanidium thiocyte-phenol-chloroform extraction method with TRIzol reagent (Invitrogen, Carlsbad, CA, USA) following the manufacturer’s protocol. RNA quantification was performed using a NanoDrop spectrophotometer (NanoDrop ND-1000 UV-Vis Spectrophotometer, Wilmington, DE, USA). Subsequently, RNA was stored at -20°C. For qRT-PCR, 1 μg of total RNA was reverse-transcribed into single stranded cDNA at 85°C for 3 min (denaturation), at 44°C for 60 min (annealing) and at 92°C for 10 min (reverse transcriptase inactivation) using the Transcriptor High Fidelity cDNA Synthesis Kit (Roche Diagnostics, West Sussex, UK). qRT-PCR analysis was performed in a total reaction mix of 20 μl per well using a LightCycler 480 SYBR Green I Master (Roche Diagnostic, Mannheim, Germany). Primers are listed in Table 1. Each reaction was run in duplicate for each sample and primer pair under the following conditions: denaturation at 95°C for 5 min, 45 cycles of denaturation (95°C, 10 sec), annealing (60°C 15 sec) and elongation (72°C 10 sec). Expression levels of MCP-1 were determined using a LightCycler 480 (Roche Diagnostics, Mannheim, Germany) and normalized against the β-actin gene expression in each sample (ΔΔC_T_-method).

**Table 1:** Gene-specific primer sequences.

Gene	Primer sequence	Product size (bp)
MCP-1 fw	CCAGAAACCAGCCAACTCTC	70
MCP-1 rev	GCGTGACAGAGACCTGCATA	
β-actin fw	TTGCTGACAGGATGCAGAAG	108
β-actin rev	TAGAGCCACCAATCCACACA	

### Western blot

Western blotting (WB) from fresh frozen whole lungs was performed as previously described [16,17]. In brief, defrosted lungs were quickly homogenized and proteins were isolated in lysis buffer (25 mM Tris-HCL, 50 mM NaCl, 5 mM MgCl_2_, 1 mM EDTA, 1% NP-40, 10% glycerol and 1% protease inhibitor cocktail). After determination of total protein concentration, equal amounts of protein (200 μg) were denatured in Laemmli sample buffer (Sigma-Aldrich Ireland Ltd., Wicklow, Ireland) and protein separation was performed by gel electrophoresis using precast 10% SDS polyacrylamide gels (NuPage Novex Bis-Tris gels, Invitrogen, Carlsbad, CA, USA) in NuPage MES SDS running buffer (Invitrogen, Carlsbad, CA, USA). Proteins were then transferred to 0.45 μm nitrocellulose and blots were blocked overnight in 3% BSA in PBST. Determination of MCP-1 was carried out using a primary antibody against MCP-1 (goat polyclonal, sc-1785; dilution 1:500, Santa Cruz Biotechnology Inc, Santa Cruz, CA, USA). After overnight incubation at 4°C (shaking), blots were extensively washed in PBST for 4 h, incubated with the secondary antibody, and washed again. Finally, the PIERCE chemiluminescence kit (Thermo Fisher Scientific, Dublin, Ireland) was used for detection. Equal loading of electrophoresis gels was controlled by Bradford assay (200 μg total protein), confirmed by coomassie staining of the gel and β-actin staining of the stripped membranes (S1 and S2 Figs). For semiquantitative analysis, protein band intensities were normalized for loading control using β-actin (ab8227, 1:5000, Abcam plc, Cambridge, UK) of stripped membranes.

### Enzyme-linked immunosorbent assay

Fetal serum was evaluated for MCP-1 concentration using the Invitrogen MCP-1 Rat Instant Enzyme-linked immunosorbent assay (ELISA) Kit (BMS631INST, Thermo Fisher Scientific, Dublin, Ireland) according to the manufacturer’s instructions. The results were measured at 450 nm with a Bio Tek Synergy^T^ Mx microplate reader (Bio Tek, Winooski, VT, USA) immediately after adding the stop solution. Experiments were carried out in duplicate for each data point.

### Immunohistochemistry

For immunohistochemistry (IHC), formalin fixed left lungs were washed overnight in PBS at 4°C, embedded in OCT Mounting tissue (VWR International, Leuven, Belgium) and stored at -80°C. Transversal sections of 10 μm thickness were cut from frozen blocks and placed on SuperFrost Plus slides (VWR International, Leuven, Belgium). Sections were then washed in PBS and underwent cell membrane permeabilization for 20 min at room temperature using 1% Triton X-100 in PBS. After washing, sections were preincubated with blocking solution for 30 min (10% bovine serum, 0.3 M glycine) followed by incubation with primary antibodies against MCP-1 (goat polyclonal, sc-1785; dilution 1:100 in PBST, Santa Cruz Biotechnology Inc, Santa Cruz, CA, USA) and smooth muscle actin (SMA, mouse monoclonal, M0851, 1:200 dilution in PBST, DAKO Diagnostics Ireland Ltd., Dublin, Ireland) overnight at 4°C. Corresponding secondary antibodies (donkey anti-goat Alexa 555 –A21432, goat anti-mouse Alexa 488 –A11029, Invitrogen, USA) were applied after washing at room temperature for 30 min. Again, sections were washed, counterstained with DAPI (1:1000 in PBST, Roche, Mannheim, Germany), mounted and coverslipped with Sigma Mounting medium (Sigma Aldrich, USA). For analysis, a Zeiss LSM 700 confocal microscope (Carl Zeiss MicroImaging, Jena, Germany) was used.

For immunohistochemistry of ED1+ cells (monocytes/macrophages), dewaxed paraffin sections of lungs were used. Detection of macrophages was performed with a mouse monoclonal antibody to CD68 (anti-rat CD68, clone ED1, MCA 341 R, AbD Serotec, Bio-Rad, Hercules, CA 94547, USA). Slides were incubated with the primary antibody for 1 h at 21°C. As secondary antibody, a biotin-SP-conjugated AffiniPure goat-anti-mouse (Jackson Immunoresearch, West Grove, PA, USA, 115-065-166) was applied for an incubation time of 30 min at 21°C. Immunostaining was done with a routine method using alkaline phosphatase streptavidin-biotin (Vector Laboratories Inc., Burlingame, CA, USA, S-5100) and as chromogen Fast Red (Fast Red substrate pack, BioGenex, Fremont, CA, USA, HK182-5K). The slides were finally counterstained with Mayer’s hematoxylin (Linaris Biologische Produkte GmbH, Wertheim-Bettingen, Germany, EGH3411). For evaluation of the number of monocytes/macrophages, ED-1 stained sections were blinded and randomized. Perivascular ED1+ monocytes/macrophages were counted in four representative, non-overlapping high-power fields per section by two independent investigators.

### Quantification of pulmonary vascular smooth muscle layer thickness and MCP- 1 staining intensity

Based on immunohistochemical SMA staining, a semi quantitative analysis of the pulmonary vascular smooth muscle layer thickness was performed. Therefore, the thickness of SMA positive pulmonary vessels was measured at three positions using the distance tool of ZEN 2.3 Software (Zeiss, Jena, Germany) in 6 representative microscopic photographs per group. Accordingly, perivascular MCP-1 intensity was assessed using Zen Software. Comparability was enabled on the basis of a standardized staining and scanning protocol including identical exposure times as well as normalization against the corresponding negative controls for each microscopic photograph.

### Statistical analysis

Data are represented as mean ± standard deviation (SD). After testing for normal distribution by Kolmogorov-Smirnov, differences between groups were evaluated by *t*-test and ANOVA with posthoc Tukey´s multiple comparisons test for parametric samples. Statistics were performed by OriginPro 8.1 (Additive GmbH, Friedrichsdorf, Germany), GraphPad Prism 7.04 (La Jolla, CA, USA) and Statistical Package for the Social Science (V24). Significance level was set as p<0.05.

## Results

### MCP-1 mRNA and protein expression is increased in nitrofen-induced CDH

**Fig 1:**
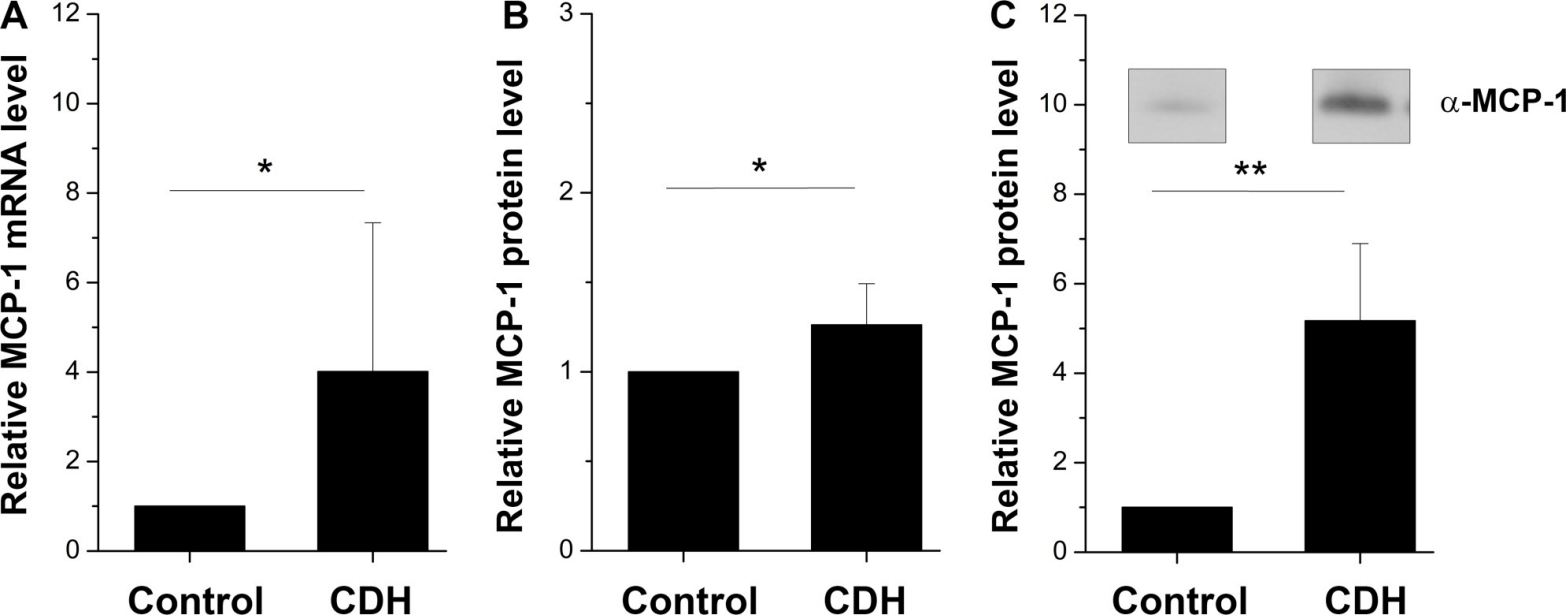
MCP-1 mRNA and protein expression. MCP-1 mRNA expression levels were significantly increased in nitrofen-exposed lungs compared to normal tissue as shown by qRT-PCR (**A**: 4.0 fold increase over control, p = 0.04). Increased systemic MCP-1 serum levels were observed in the CDH group compared to control by ELISA (**B**, 1.3 fold increase over control, p = 0.01). Western blotting analyses confirmed these findings by revealing significantly increased MCP-1 protein levels in CDH lung tissue (**C**, 5.2 fold increase over control, p = 0.004). Statistical analysis by Student´s *t*-test, *p<0.05, **p<0.01.

**Fig 2:**
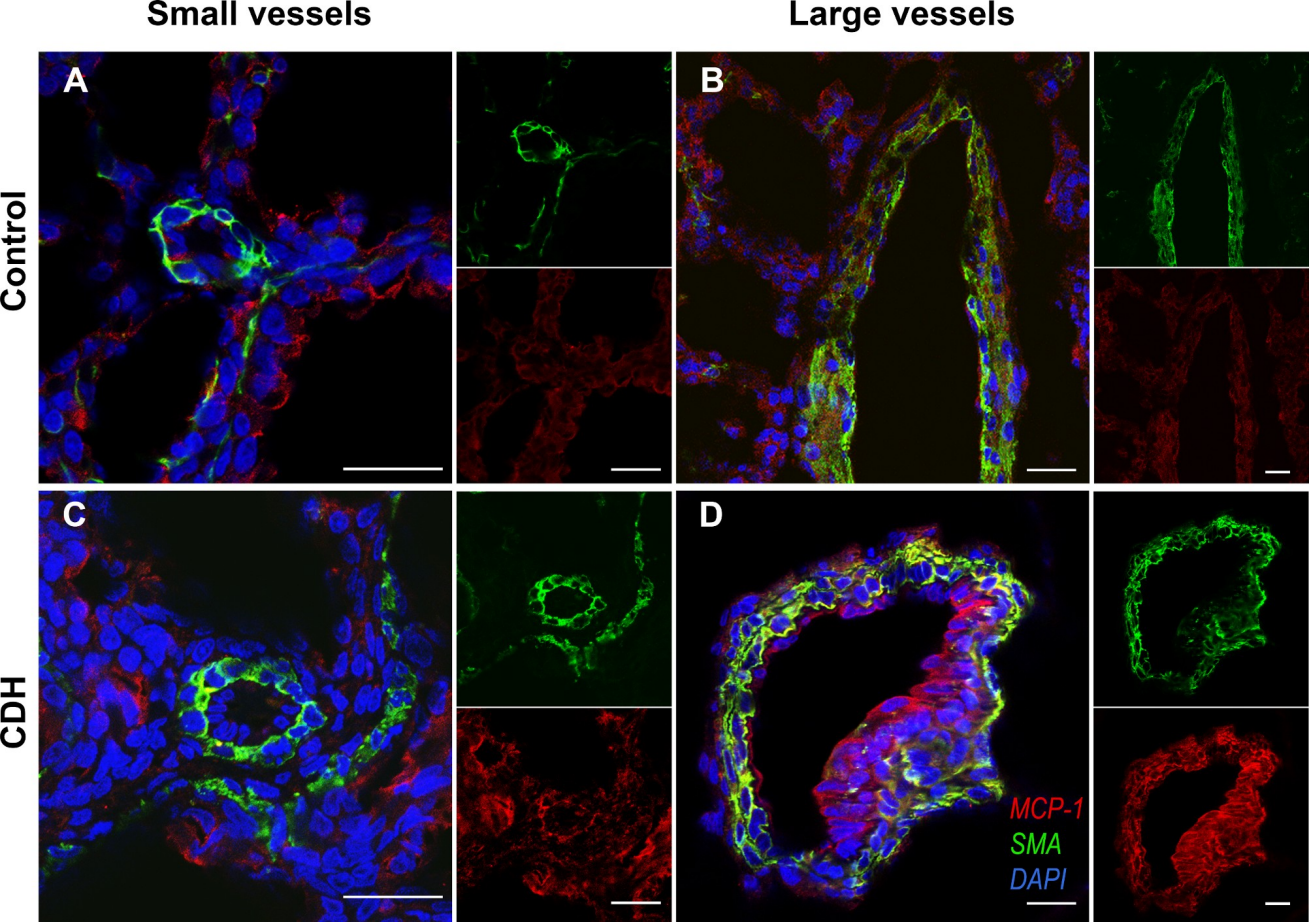
Pulmonary MCP-1 expression. Immunofluorescence evaluation of MCP-1 and SMA demonstrated markedly increased medial and adventitial thickness of small (**A**, **C**) and large (**B**, **D**) pulmonary vessels in CDH lung tissue compared to controls. Additionally, immunoreaction of MCP-1 and SMA was augmented around pulmonary arteries in CDH lung tissue (**C**, **D**). Scale bar 30 μm.

Nitrofen-exposed lung tissue revealed significantly increased MCP-1 mRNA levels compared to control lung as shown by qRT-PCR (Fig 1A; 4.0 fold increase over control; p = 0.04). ELISA (Fig 1B; 1.3 fold increase over control; p = 0.01) revealed an increased systemic MCP-1 serum concentration and WB (Fig 1C; 5.2 fold increase over control; p = 0.004) confirmed that the increased MCP-1 transcripts in samples of CDH lungs resulted in significantly increased MCP-1 protein levels compared to control lungs (S1 Fig). Additionally, a generally strong immunoreactivity of MCP-1 in CDH lung sections indicated elevated MCP-1 levels (Fig 2).

### Vascular remodeling in nitrofen-induced CDH is accompanied by increased pulmonary MCP-1 protein expression and pulmonary monocyte/macrophage influx

Medial and adventitial thickness of small and large pulmonary vessels was markedly increased in CDH lungs compared to controls. The observed vascular remodeling was accompanied by a strong perivascular immunoreaction of MCP-1 around small as well as large pulmonary vessels in CDH lungs, as shown by SMA and MCP-1 immunostaining (Fig 2).

**Fig 3:**
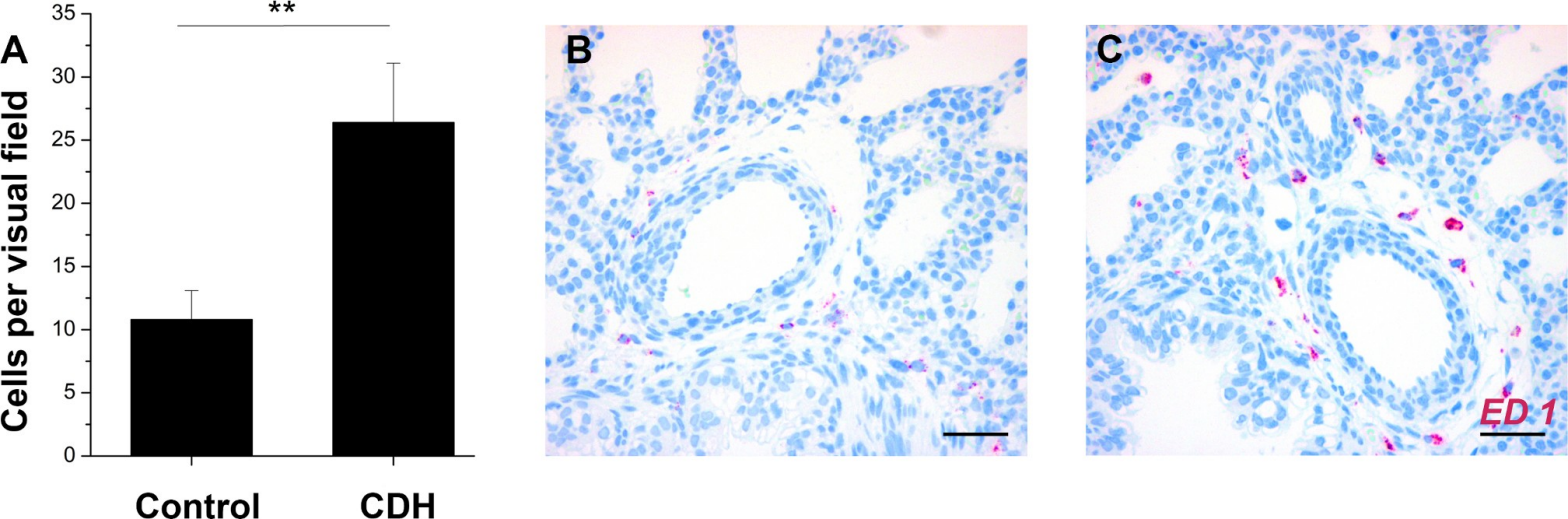
Pulmonary monocytes/macrophages. Quantification of ED1-positive cells (**A**) revealed significantly increased numbers of monocytes/macrophages in CDH lungs. Immunofluorescence showed an accumulation of ED1-positve cells around pulmonary vessels in nitrofen-exposed CDH lungs (**C**) compared to controls (**B**). Statistical analysis by Student´s *t*-test, *p<0.05, **p<0.01, p***<0.001, Scale bar 50 μm.

To assess the effect of different MCP-1 levels on monocytes/macrophages, immunohistochemistry and quantification of ED1-positive cells on immunostained sections were performed. Both procedures demonstrated significantly increased levels of monocytes/macrophages in lungs of nitrofen-exposed animals (Fig 3).

### Effects can be reversed by prenatal application of rosiglitazone

The effect of prenatally administered rosiglitazone was investigated by double immunostaining (SMA, MCP-1) as well as WB (MCP-1) as described for experimental set 1. SMA as well as MCP-1 were observed in an alleviated pattern, which was similar to those of control sections after prenatal treatment with rosiglitazone in nitrofen-exposed dams.

**Fig 4:**
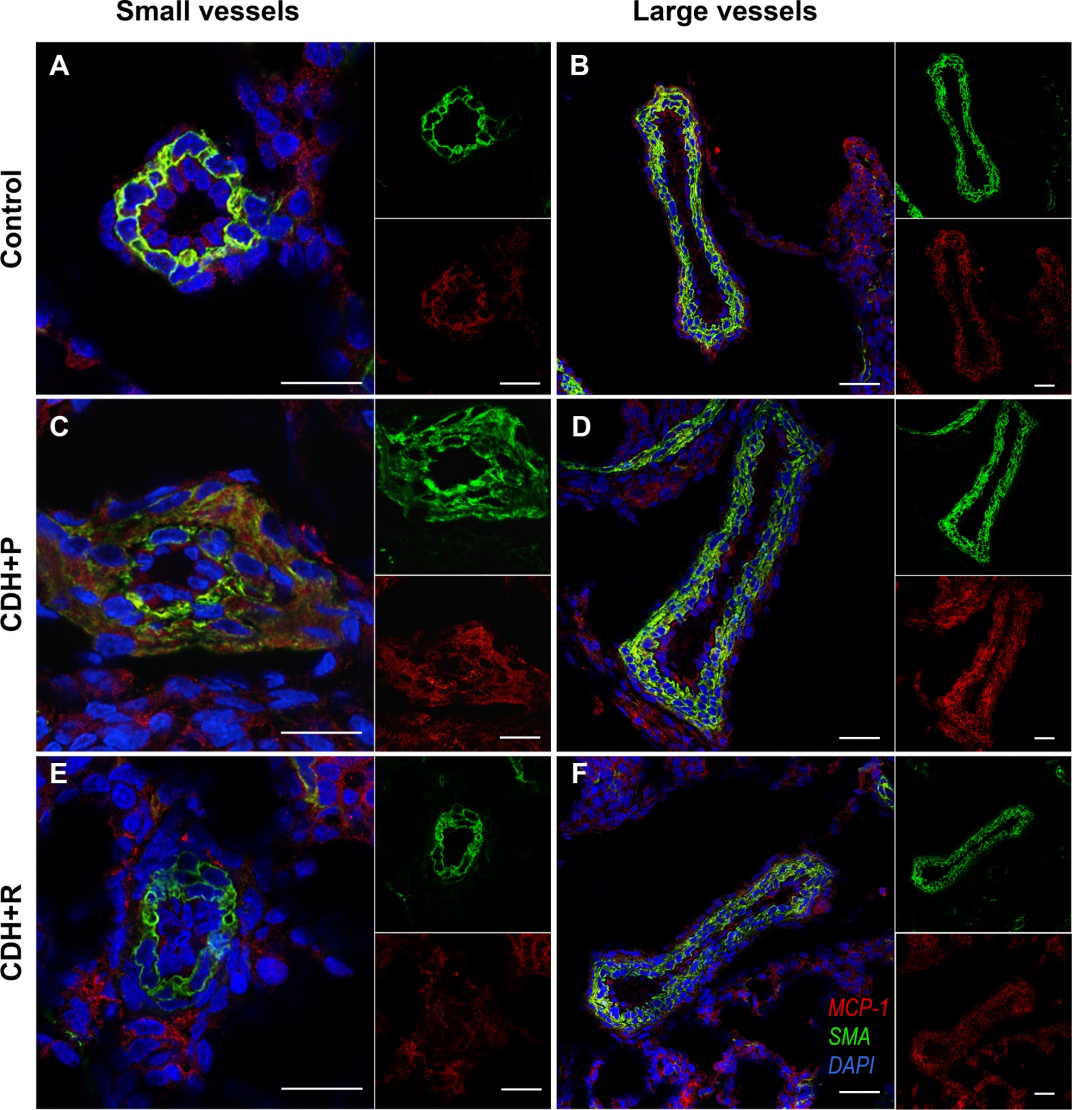
Pulmonary MCP-1 expression after prenatal treatment with rosiglitazone. After prenatal treatment with rosiglitazone (CDH+R; **E**, **F**), no increase in thickness of pulmonary vessels as well as of MCP-1 and SMA immunoreactivity could be observed in CDH lungs compared to controls (control; **A**, **B**). Treatment with placebo only resulted in the typical medial and adventitial changes as well as increased immunoreactivity of MCP-1 and SMA in the pulmonary vasculature of CDH fetuses (CDH+P; **C**, **D**). Scale bar 30 μm.

Significantly increased thickness of pulmonary vessels of all sizes was confirmed in CDH lung tissue from rats treated with placebo only (CDH+P, p = 0.001) and could not be identified in fetuses prenatally treated with rosiglitazone on D18 and D19 (CDH+R, Fig 4, S3A Fig).

**Fig 5:**
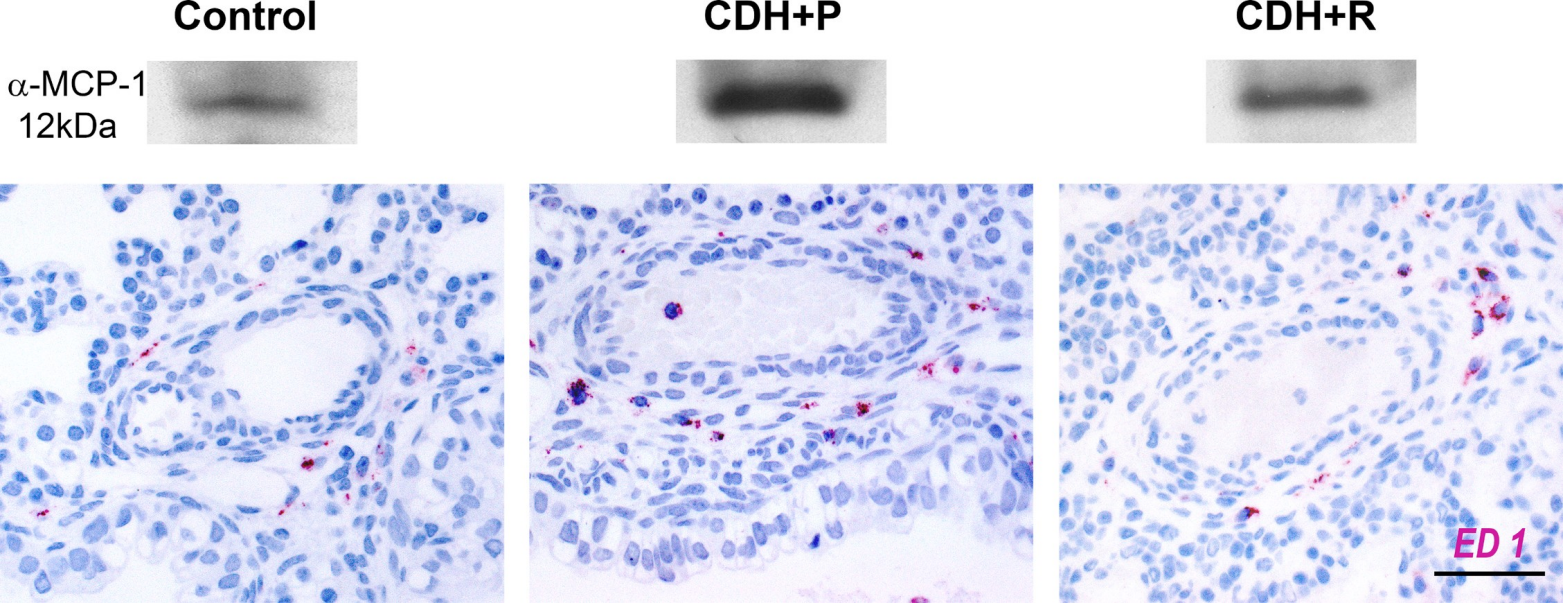
Pulmonary MCP-1 expression and monocytes/macrophages after prenatal treatment with rosiglitazone. MCP-1 protein levels were decreased in lungs of CDH fetuses treated with rosiglitazone (CDH+R) compared to CDH fetuses exposed to placebo only (CDH+P), as demonstrated by western blotting. This was accompanied by a significantly reduced number of pulmonary monocytes/macrophages, shown by ED1 immunohistochemistry. Scale bar 50 μm.

Overall as well as perivascular MCP-1 protein expression were significantly decreased in lung tissue of rosiglitazone-treated animals with CDH compared to lungs of placebo-treated CDH animals as shown by western blotting (Fig 5) and immunohistochemistry (S3A and S3B Fig, p = 0.018).

ED1 immunohistochemistry revealed significantly reduced numbers of pulmonary monocytes/macrophages in sections of rosiglitazone-treated animals compared to placebo-treated CDH animals (19.8±4 vs. 26.4±-5.0, p = 0.006; Fig 5).

## Discussion

PH is one of the key determinants of outcome in patients with CDH. It is characterized by increased pressure in the pulmonary artery circulation resulting from a significant vascular remodeling of small and large pulmonary vessels [6]. Histologically, a medial and adventitial thickening of pulmonary arteries of all sizes has been described in human specimen and can be reliably reproduced in the nitrofen CDH model [18–22]. In the present study, these specific features of vascular remodeling have been confirmed: We observed a relevant thickening of pulmonary vessels with increased layers of vascular smooth muscle cells (SMCs) in fetal rat lungs with CDH compared to controls. However, the molecular background of vascular remodeling observed in nitrofen-induced CDH remains elusive.

Normal pulmonary vasculogenesis is dependent on a crucial balance between proliferation and apoptosis of SMCs [23,24]. Besides its key function in regulation of the migration and infiltration of monocytes/macrophages, the chemokine MCP-1 has recently been attributed an important role as a potent inductor of SMC proliferation [25]. In this regard, increased MCP-1 levels have been shown to be associated with the development of severe PH due to increased vascular remodeling in neonatal and preterm lungs of humans and in rodent models [8,13,26–30]. However, the prenatal relevance of pulmonary MCP-1 expression especially in CDH has not been investigated to date.

We previously reported on increased pulmonary MCP-1 mRNA expression in fetal lungs of nitrofen-induced CDH rats [15]. In the present study, we proved that the increased pulmonary MCP-1 mRNA transcripts observed in CDH were translated to the protein level. Increased MCP-1 protein expression was predominantly localized perivascular, suggesting a possible association of MCP-1 on the prenatal pulmonary vascular development. It remains unclear if this observation represents the cause or the result of prenatal vascular remodeling in experimental CDH.

MCP-1 is produced by alveolar epithelial, endothelial, SMCs, and monocytes/macrophages after exposition with acute phase proteins, bacterial or viral products [26,31]. The activation of perivascular monocytes/macrophages by increased MCP-1 expression has been shown to result in endothelial dysfunction and SMC proliferation. Based on rodent and large animal models, increased MCP-1 expression has been suggested to contribute to a pro-inflammatory phenotype [10,25,32–34]. This pro-inflammatory phenotype is maintained due to aberrant cellular cross-talk between mesenchymal cells and macrophages and subsequently promotes transition to chronic non-resolving inflammation and vascular remodeling [25].

To evaluate the impact of increased pulmonary MCP-1 expression in our model, we investigated the respective target cells. In the present study, increased perivascular MCP-1 protein expression was accompanied by significantly enhanced pulmonary infiltration of ED1-positive monocytes/macrophages in fetal rat lungs with CDH compared to controls. This enhanced perivascular monocyte/macrophage infiltration suggests a relevant impact of the observed MCP-1 protein expression pattern in fetal CDH lungs, thus contributing to extensive vascular remodeling by maintaining a pro-inflammatory phenotype.

Besides the induction of an anti-inflammatory signaling cascade, the transcription factor peroxisome proliferator-activated receptor γ (PPARγ) has been attributed a key role in physiological pulmonary angiogenesis during the late stages of fetal lung development together with the bone morphogenetic protein receptor 2 (BMPR2) [9,11,16,35–37]. PPARγ/BMPR2 signaling is essential for the modulation of differentiation, proliferation, and the fibrous matrix production of both endothelial and SMCs by controlling an antiproliferative signaling pathway [9,16,36]. Disruption of this signaling cascade has been shown to result in uncontrolled SMC proliferation significantly contributing to PH in humans and experimental settings [9,35,36,38]. In contrast, activation of the PPARγ/BMPR2 signaling pathway has been suggested to prevent vascular remodeling ensuring a normal vasculogenesis [9,11,37]. MCP-1 expression and vascular remodeling can be inhibited by the PPARγ-agonist rosiglitazone (thiazolidinedione) [9,39]. However, prenatal effects of rosiglitazone on the pulmonary vasculature of fetuses with CDH have not been evaluated to date. In the present study, we show for the first time that prenatal application of rosiglitazone on D18 and D19 reduced MCP-1 expression, monocyte/macrophage infiltration and vascular remodeling in fetuses with CDH compared to placebo-treated animals. These data clearly show that prenatal activation of the PPARγ/BMPR2 signaling pathway attenuates the previously described features of vascular remodeling in experimental CDH. We therefore suggest that modulation of this signaling cascade represents a potential target for prenatal attenuation of PH in CDH.

Limitations of our study include the lack of data on postnatal outcome after prenatal treatment with rosiglitazone. Survival studies of rats after nitrofen-induction of CDH do not meet up-to-date ethical standards. Due to the severity of PH and hypertension in this animal model, the majority of the neonatal rats die immediately after birth.

To evaluate the relevance of our findings regarding a translation from bench to bedside, the next step will be to investigate whether the PPARγ/BMPR2 signaling is altered in human CDH as well and may therefore have a significant impact on future treatment strategies.

## Conclusions

Increased pulmonary MCP-1 expression in fetal rat lungs with CDH has a relevant impact on prenatal pulmonary monocyte/macrophage infiltration and vascular remodeling. Prenatal treatment with rosiglitazone has the potential to attenuate activation of pulmonary MCP-1, pulmonary monocyte influx, and vascular remodeling in experimental CDH. These results provide a basis for future research on prenatal immunomodulation as a novel treatment strategy to decrease secondary effects of PH in CDH.

## Supporting information

S1 Fig Pulmonary MCP-1 protein expression. Western blotting result of experimental set 1 show an increased MCP-1 protein expression in CDH lung tissue compared to controls (**A**). Equal loading of electrophoresis gels was controlled by Bradford assay and confirmed by beta-actin staining of the stripped membranes (**B**).
(TIF)

S2 Fig Pulmonary MCP-1 protein expression after treatment with rosiglitazone. Western blotting result of experimental set 2 show an increased MCP-1 protein expression in CDH lung tissue from rats treated with placebo (CDH+P) compared to controls and CDH lungs from rosiglitazone-treated fetuses (CDH+R) (**A, B**). Equal loading of electrophoresis gels was controlled by Bradford assay and confirmed by Coomassie staining of the gel (**A1, B1**).
(TIF)

S3 Fig Vascular smooth muscle layer thickness and perivascular MCP-1 expression. (**A**) The relative vascular smooth muscle layer thickness was significantly increased in CDH lung tissue from rats treated with placebo only (CDH+P) compared to controls (p = 0.001) and fetuses prenatally treated with rosiglitazone on D18 and D19 (CDH+R, p = 0.008).
(**B**) Perivascular MCP-1 protein expression was significantly decreased in lung tissue of rosiglitazone-treated animals with CDH (CDH+R, p = 0.018) compared to lungs of placebo-treated CDH animals (CDH+P). Statistical analysis by ANOVA with posthoc Tukey´s test, *p<0.05, **p<0.01, p***<0.001.
(TIF)
