# Supplementary figures and images for: Prenatal treatment with rosiglitazone attenuates vascular remodeling and pulmonary monocyte influx in experimental congenital diaphragmatic hernia
Source: PLoS One. 2018 Nov 12;13(11):e0206975. doi: 10.1371/journal.pone.0206975 (PMC6231640; doi:10.1371/journal.pone.0206975)

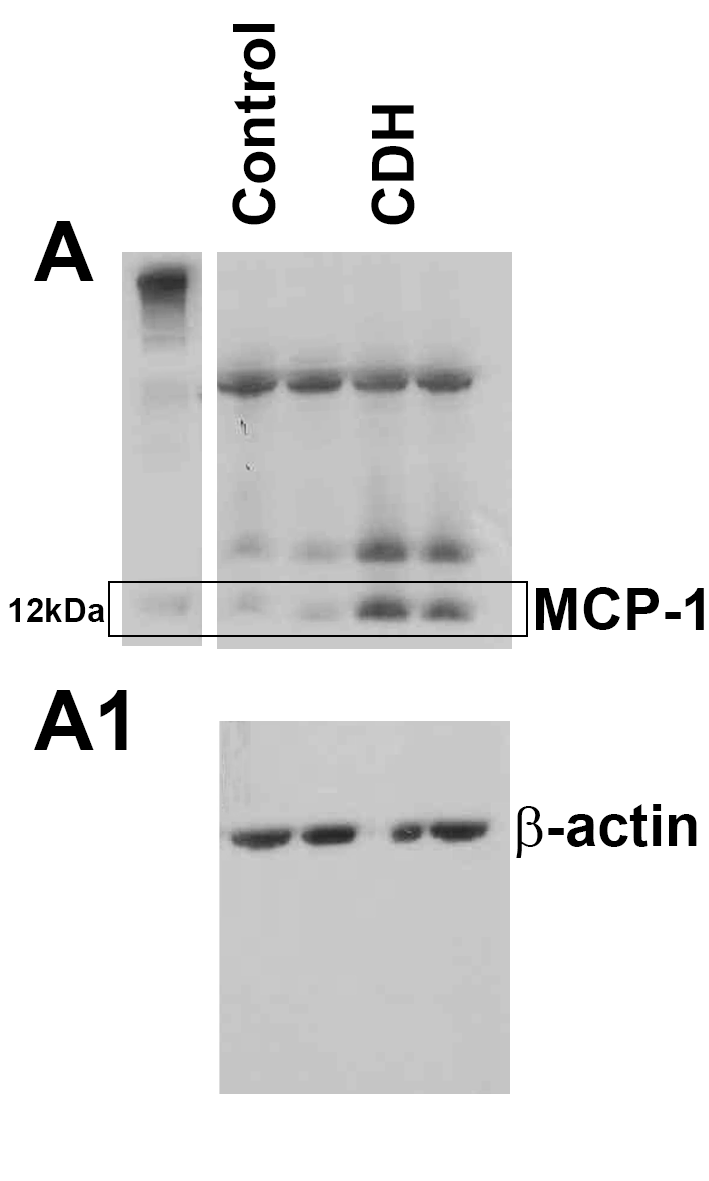

Supplement: S1 Fig — Western blotting result of experimental set 1 show an increased MCP-1 protein expression in CDH lung tissue compared to controls (A). Equal loading of electrophoresis gels was controlled by Bradford assay and confirmed by beta-actin staining of the stripped membranes (B). (TIF) [file pone.0206975.s001.tif]

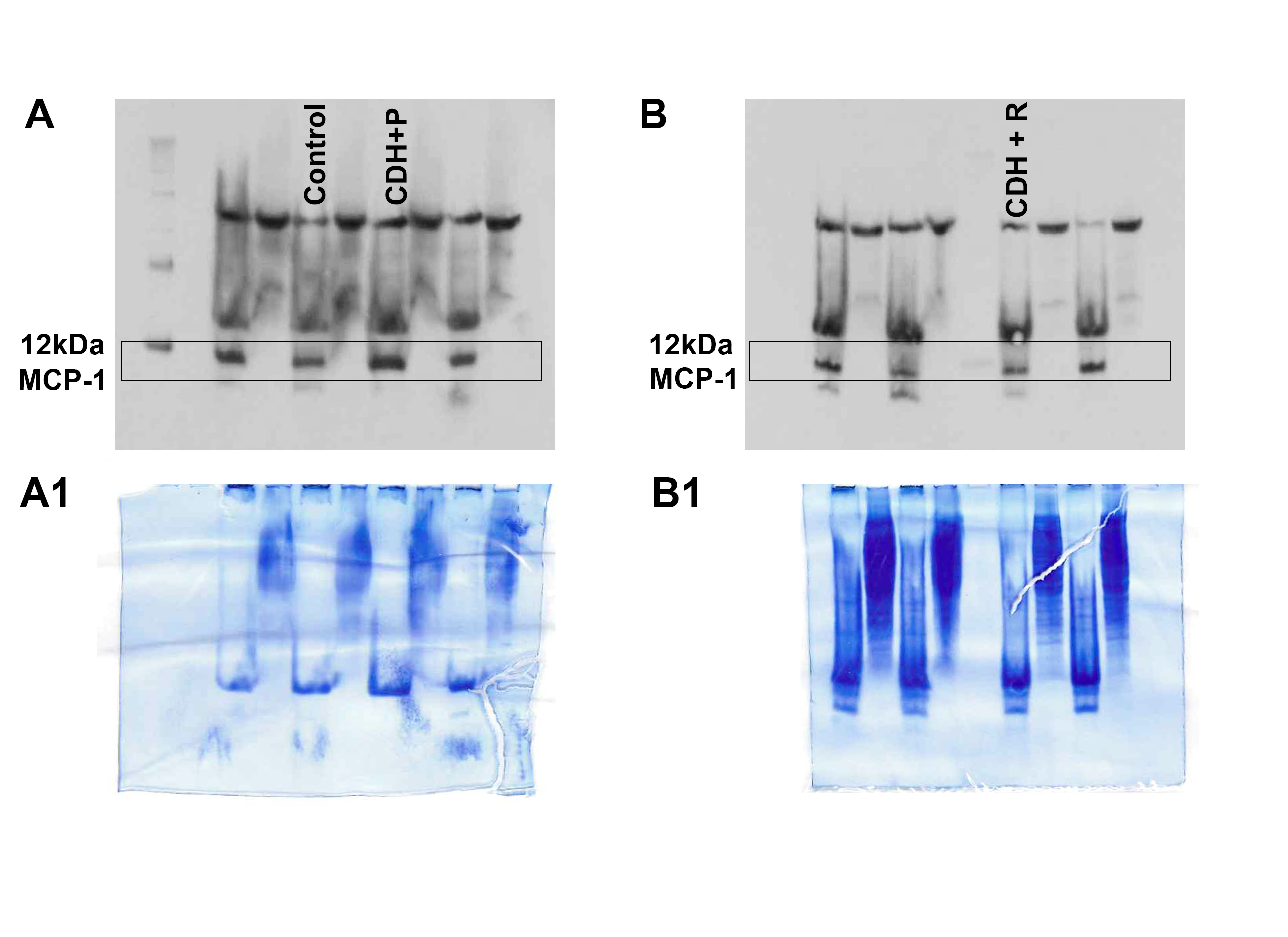

Supplement: S2 Fig — Western blotting result of experimental set 2 show an increased MCP-1 protein expression in CDH lung tissue from rats treated with placebo (CDH+P) compared to controls and CDH lungs from rosiglitazone-treated fetuses (CDH+R) (A, B). Equal loading of electrophoresis gels was controlled by Bradford assay and confirmed by Coomassie staining of the gel (A1, B1). (TIF) [file pone.0206975.s002.tif]

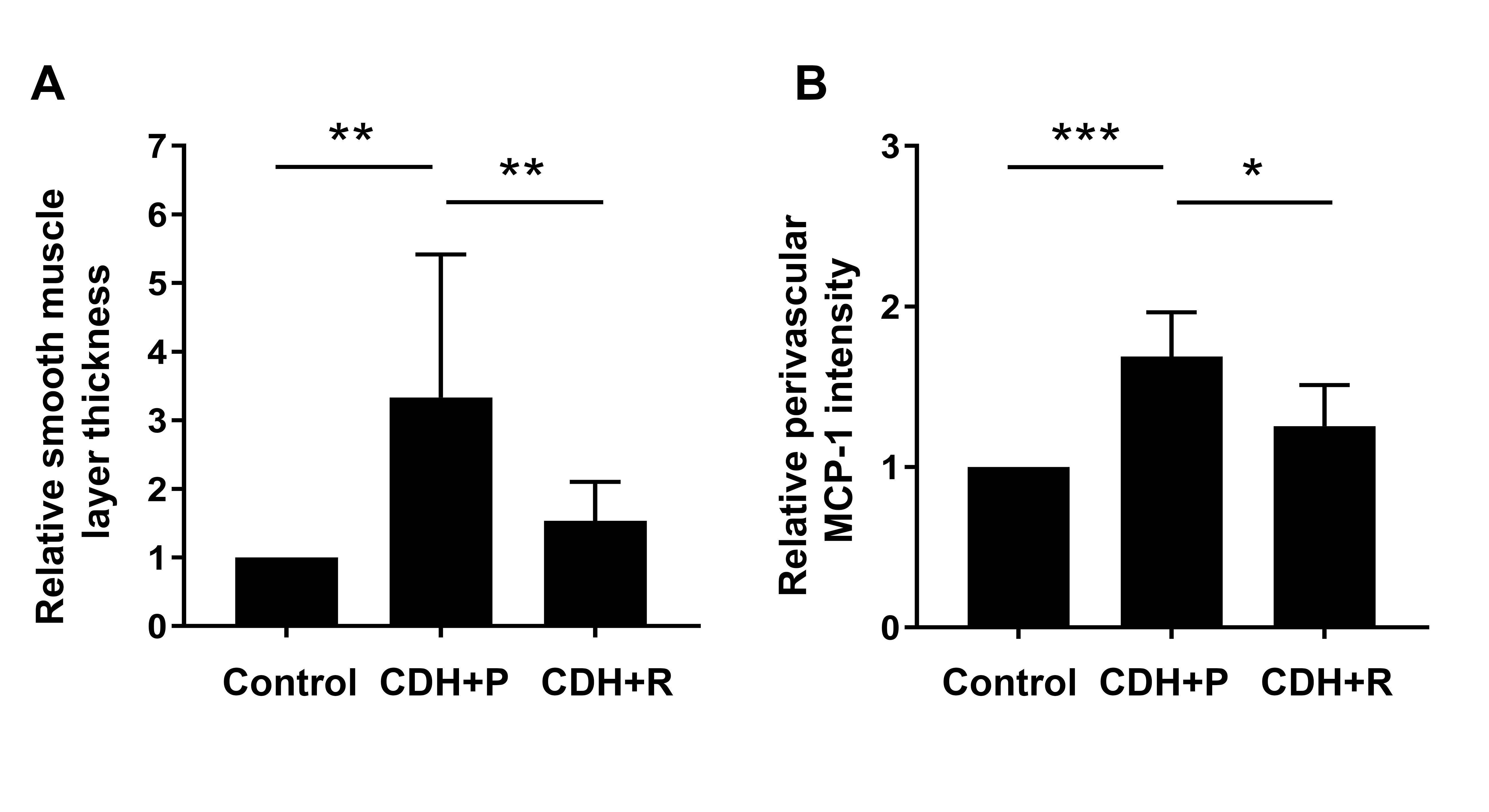

Supplement: S3 Fig — (A) The relative vascular smooth muscle layer thickness was significantly increased in CDH lung tissue from rats treated with placebo only (CDH+P) compared to controls (p = 0.001) and fetuses prenatally treated with rosiglitazone on D18 and D19 (CDH+R, p = 0.008). (B) Perivascular MCP-1 protein expression was significantly decreased in lung tissue of rosiglitazone-treated animals with CDH (CDH+R, p = 0.018) compared to lungs of placebo-treated CDH animals (CDH+P). Statistical analysis by ANOVA with posthoc Tukey´s test, *p<0.05, **p<0.01, p***<0.001. (TIF) [file pone.0206975.s003.tif]
